# Supplementary figures and images for: An update to Hippocampome.org by integrating single-cell phenotypes with circuit function in vivo
Source: PLoS Biol. 2021 May 6;19(5):e3001213. doi: 10.1371/journal.pbio.3001213 (PMC8130934; doi:10.1371/journal.pbio.3001213)

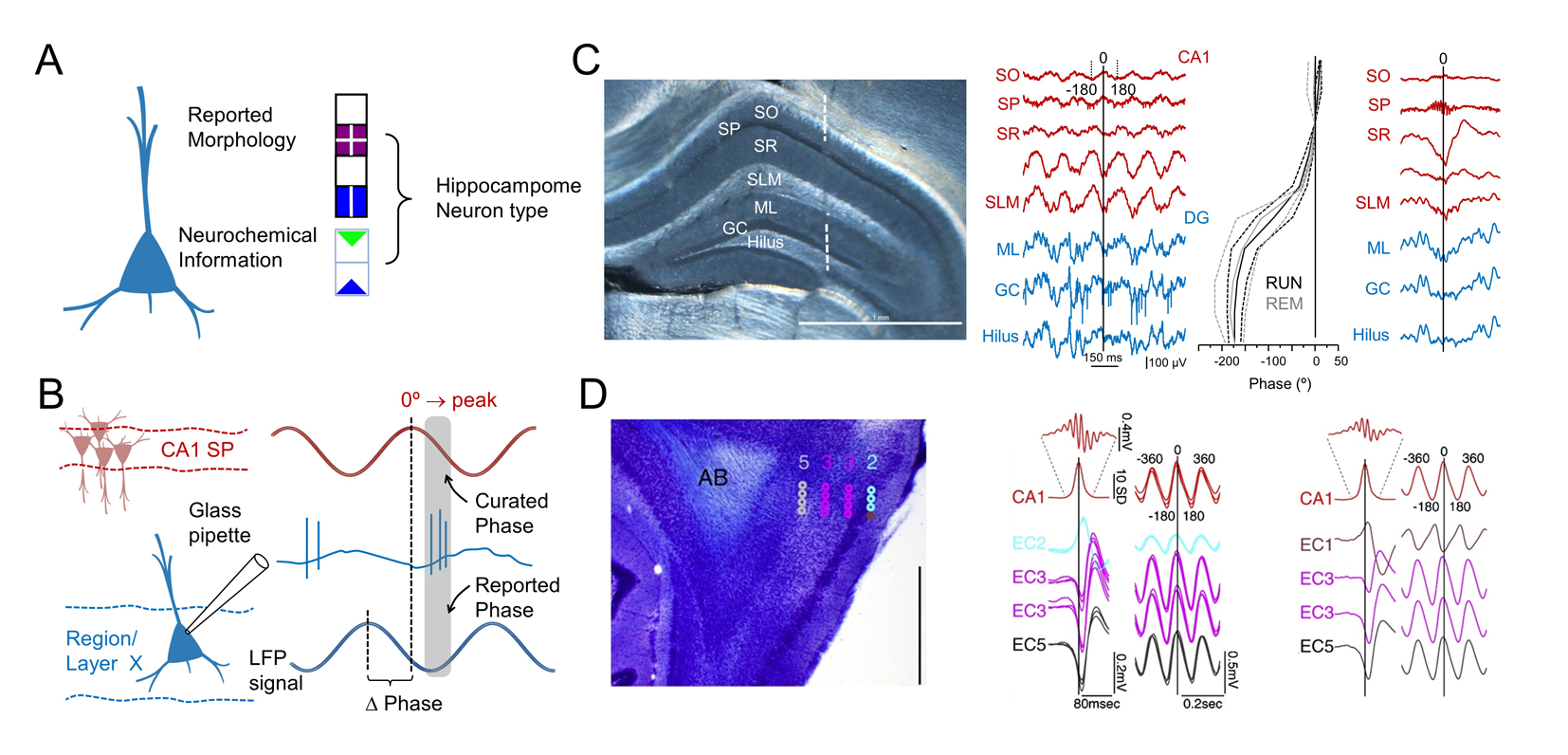

Supplement: S1 Fig — (A) Method to ascribe a particular cell to a Hippocampome.org neuron type. (B) Equivalence between reported theta phases of single cells in a given region/layer and the reference theta clock at CA1. Note our convention of 0° at the CA1 SP theta peak. (C) Left: Coronal section of the dorsal hippocampus showing the track of the silicon probe shank (discontinuous line) across layers. Center/right: Representative LFP traces across layers from CA1 (red) to the DG (blue) during theta oscillations and (right) SWR events. The plot shows the polarity reversal of theta cycles across layers for theta oscillations recorded during RUN and REM sleep. CA1 layers: SO, stratum oriens; SP, stratum pyramidale; SR, stratum radiatum; and SLM, stratum lacunosum moleculare. DG layers: ML, molecular layer; GC, granule cell layer; and the hilus. Scale bar 1 mm. Data from this work. (D) Left: Section of the EC showing the reconstructed tracks of the four-shank silicon probe (200 μm inter-shank distance). Numbers indicate the EC layers. Scale bar 1 mm. Right: Phase relationship between hippocampal events (SWR and theta cycles) recorded across EC layers. Note the similar phases across layers 5 to 2 and polarity reversal in EC1. Modified from Mizuseki and colleagues [39]. AB, angular bundle; DG, dentate gyrus; EC, entorhinal cortex; GC, granule cell; LFP, local field potential; ML, molecular layer; REM, rapid-eye movement; RUN, running; SWR, sharp-wave ripple. (TIF) [file pbio.3001213.s001.tif]

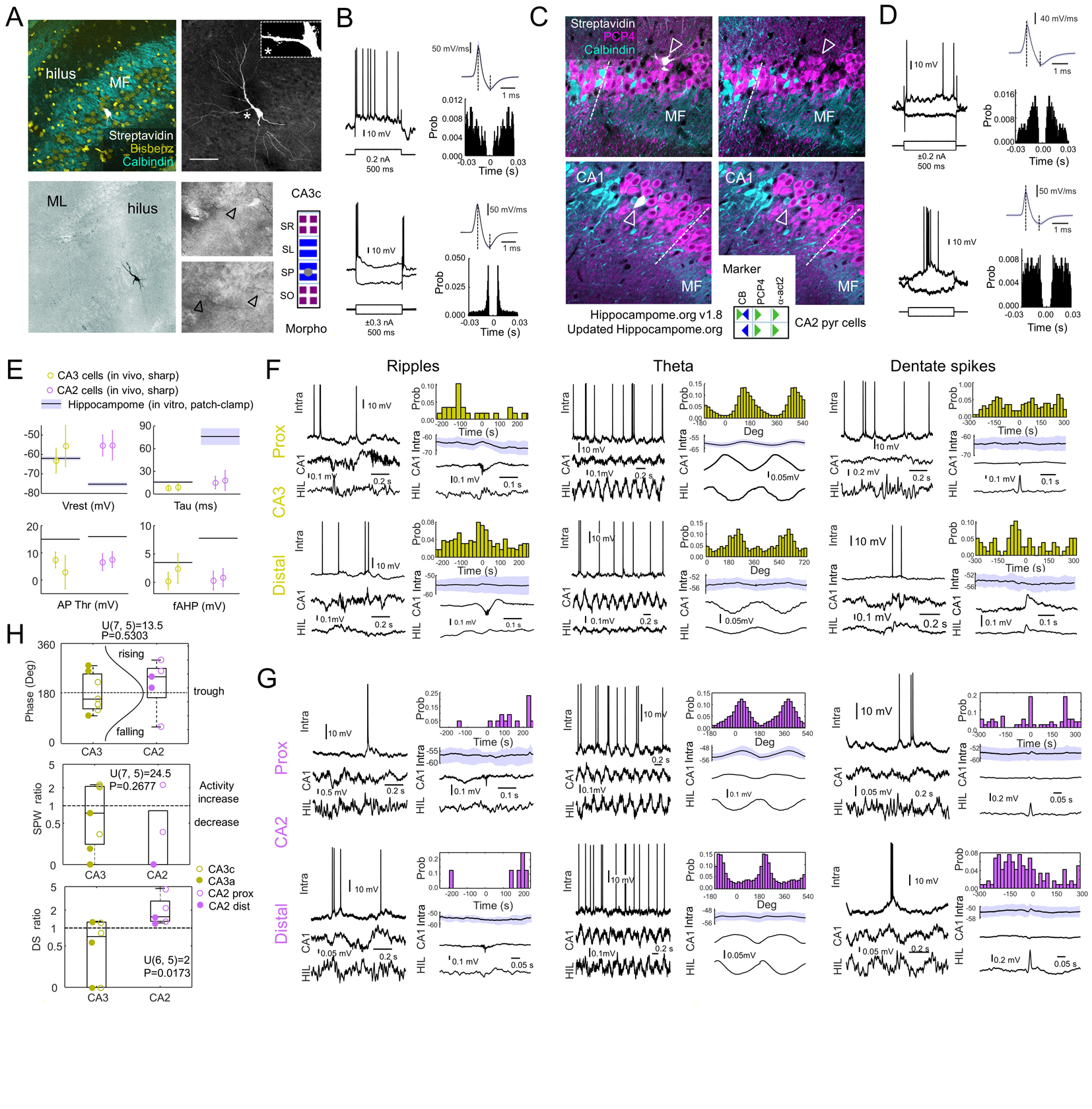

Supplement: S2 Fig — (A) Proximal CA3 pyramidal cell recorded from the CA3c region. Note thorny excrescences characteristics of this cell type. Another CA3c cell recorded near the border with CA3b is shown at bottom, and their apical dendrites indicated by arrowheads. The Hippocampome.org morphological encoding is shown at right (purple: axon and dendrites in layer; blue: dendrites in layer). (B) Firing properties of the 2 CA3 pyramidal cells shown in A in response to current pulses are consistent with nonadapting firing and silence preceded by transient slow-wave bursting (TSWB.NASP and TSWB.SLN). AP waveform and firing autocorrelogram are shown at right. (C) Examples of a proximal CA2 cell (CA2b; top) and a distal CA2 cell (CA2a; bottom). Cell identity is confirmed with immunostaining against CB and PCP4. Note that CA2 pyramidal cells are all negative to CB (Table 3). See also Fernandez-Lamo and colleagues [31] for immunoreactivity against α-actinin2. The Hippocampome.org biomolecular marker encoding is updated to clarify this ambiguity (green-blue triangle: positive–negative unresolved for subtypes; green triangle: positive; blue triangle: negative). (D) Firing properties of CA2 cells shown in C. (E) Membrane biophysics properties of CA3 and CA2 cells recorded in vivo as compared with in vitro data from Hippocampome.org. (F) Firing pattern of CA3 cells during SWR, theta, and DS. (G) Firing pattern of CA2 cells during SWR, theta, and DS. (H) Group mean theta phase-locking preferences of all CA3 and CA2 pyramidal cells in the database (upper plot). Same for the SWR ratio (middle plot) and for the DS ratio (bottom plot). Data from n = 7 CA3 cells and n = 5 CA2 pyramidal cells. See Table 2. The underlying data can be found in S1 Data. AP, action potential; CB, calbindin; DS, dentate spikes; HIL, hilus; MF, mossy fiber; ML, molecular layer; NASP, non-adapting spiking; SL, stratum lucidum; SLN, silence; SO, stratum oriens; SP, stratum pyramidale; SR, stratum radiatum; SWR, sharp-wave rippl [file pbio.3001213.s002.tif]

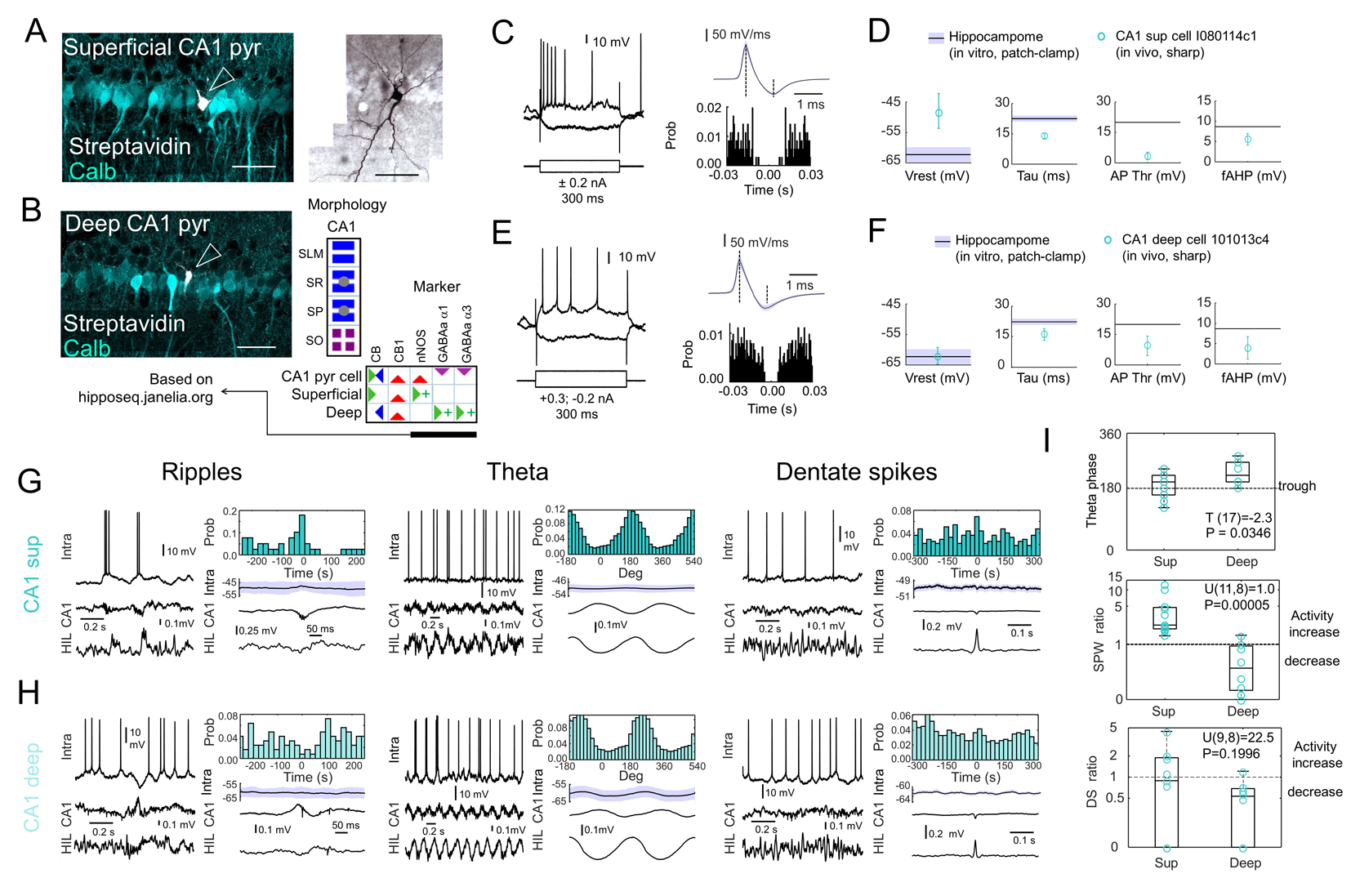

Supplement: S3 Fig — (A) Example of a superficial pyramidal cell recorded intracellularly from the dorsal CA1 region. Note location within the CB-positive sublayer. Somadendritic reconstruction after Neurobiotin processing is shown at right. (B) Example of a CB-negative deep pyramidal cell. The Hippocampome.org morphological and molecular marker encodings are shown at right. Note some unresolved expression data in v1.8 of Hippocampome.org (CA1 pyr cells), which are clarified by introducing the deep and superficial subtypes. Data on differential expression level is based on hipposeq.janelia.org (Cembrowski and colleagues [55]). The blue-green triangles indicate unclear positive–negative evidence for subtypes, red triangles for positive–negative (unresolved), magenta triangles for positive–negative (subcellular expression differences), and green triangles with pluses for differential expression favoring one subtype over the other. This latter depiction of gradients is for illustrative purposes only, as the molecular marker matrix of Hippocampome.org does not grade positive expression; however, Hippocampome.org reports information on expression gradients both in the evidential pages as well as in the Notes section of the neuron pages (Table 1). (C) Firing properties of the superficial CA1 pyramidal cell shown in A in response to current pulses consistent with ASP. The spike waveform and the firing autocorrelogram are also shown. (D) Membrane biophysics properties of superficial CA1 cells recorded in vivo as compared with in vitro data from Hippocampome.org. (E, F) Same for the deep CA1 pyramidal cell shown in B. (G) Firing pattern of the superficial CA1 pyramidal cell shown above during SWR, theta, and DS. (H) Same for the deep cell shown above. (I) Group mean theta phase-locking preferences of all CA1 pyramidal cells in the database (top plot). Same for the SWR ratio (bottom). Statistical differences between deep and superficial cells are evident (T: Student t test; U: Mann–Whitney test). [file pbio.3001213.s003.tif]

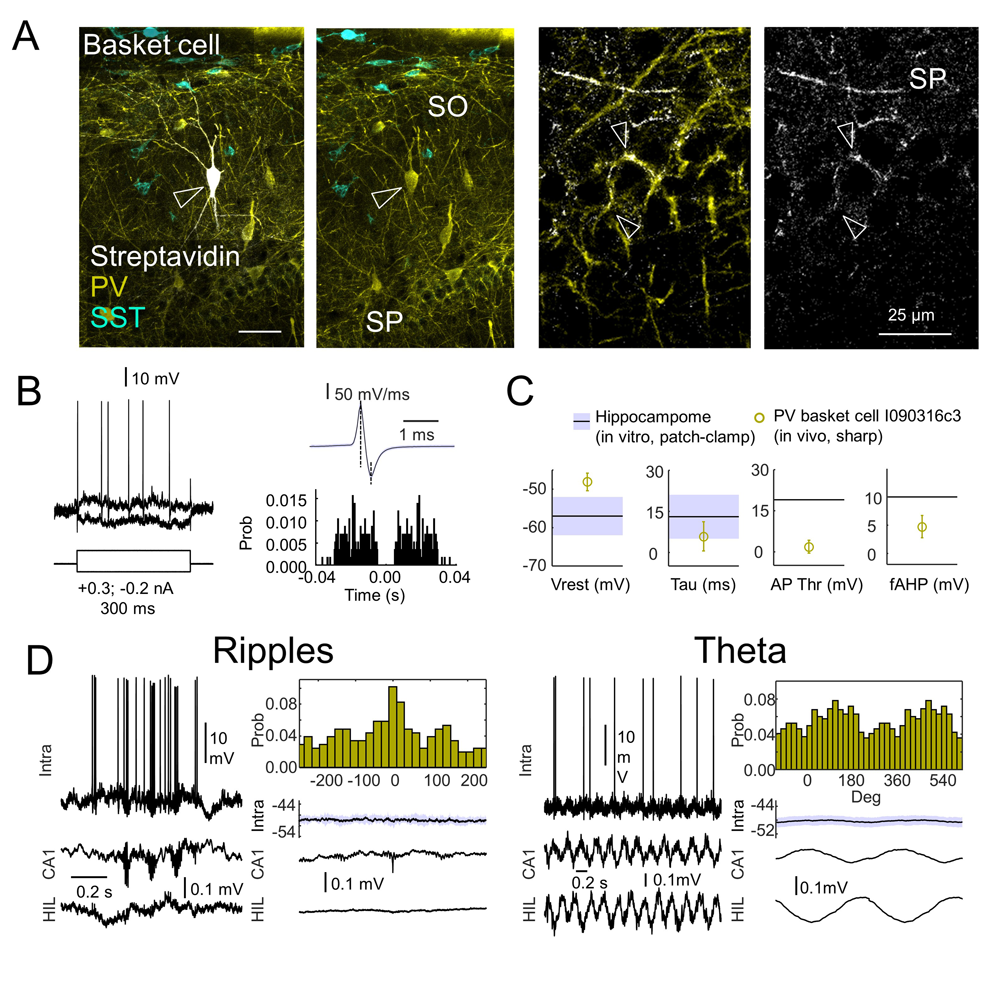

Supplement: S4 Fig — (A) Morphological and neurochemical identification of a PV basket cell recorded in vivo with sharp glass pipettes. (B) Firing properties of the PV basket cell shown in A in response to current pulses. The spike waveform and firing autocorrelogram are shown at right. (C) Membrane biophysics properties of PV basket cells recorded in vivo as compared with in vitro data from Hippocampome.org. (D) Firing pattern of the PV basket cell shown in panels A and B during SWR and theta oscillations. The underlying data can be found in S1 Data. AP, action potential; HIL, hilus; PV, parvalbumin; SO, stratum oriens; SP, stratum pyramidale; SST, somatostatin. (TIF) [file pbio.3001213.s004.tif]
