# Supplementary material for: An update to Hippocampome.org by integrating single-cell phenotypes with circuit function in vivo
Source: PLoS Biol. 2021 May 6;19(5):e3001213. doi: 10.1371/journal.pbio.3001213 (PMC8130934; doi:10.1371/journal.pbio.3001213)
Supplement: Attachment — Submitted filename: Rebuttal letter.docx [file pbio.3001213.s009.docx]

Editor’s comments: Editorially, we think the Introduction reads well. However, the use of PoE and PoK abbreviations for “pieces of evidence” and “pieces of knowledge” is distracting and creates confusion in the Results.

We thank the editor for this suggestion. We have rephrased the text to avoid the confusing use of PoE and PoK abbreviations.

More importantly, the Results section on the curated data reads more like a Methods section of how you did the curation, rather than providing clear insights into what you found from curating these data. While this likely reflects that you found a lot of data, and used it to update the Hippocampome.org website, these sections of the Results would be stronger if you could specifically provide a table and write-up of all the key findings in a systematic way (rather than describing a series of examples in a narrative).

These are excellent suggestions, which are very much aligned with revision required to address some comments by Reviewer 3. As you noticed, by explaining how curation was done we identify important knowledge gaps: unresolved heterogeneity, inconsistent recording conditions, non-standard reporting of theta phases, etc. To address your criticism and improve readability, we have substantially edited the manuscript; specifically: (i) we moved the most technical description of how we did the curation from the Results to the Methods; (ii) we reduced the overall length of narrative providing series of examples to sharpen, rather than drag, the most novel discoveries; (iii) we added a new Table 3 and accompanying paragraph at the beginning of Discussion to highlight the clear insights into what we found from the synergy of data curation and additional experiments. We chose to provide the summary table in the Discussion, rather than in the Results, to provide the requested synthesis of all the key findings in a systematic way. We felt that placing this Table at the beginning of the Results may be confusing as it would anticipate conclusions before they can be convincingly supported. If you prefer, we are open to moving this “conclusion” Table to the end of the Results or remain of course open to your further recommendations.

The later part of the Results is better, providing some useful analyses of how this expanded website can be useful to the community.

We are grateful for your and the reviewers’ constructive and positive feedback. We believe the revised manuscript is substantially strengthened and will constitute a valuable product for the readership of PLoS Biology.

Reviewer #1: This manuscript by Sanchez-Aguilera and colleagues, reports an update on the authors' ongoing efforts to develop and curate an online database (Hippocampome.org) that contains a comprehensive catalogue of hippocampal and entorhinal cortical cell types with available literature information about the firing dynamics during in vivo oscillations. Furthermore, the authors extend this knowledge with new data from in vivo recordings from hippocampal principal cells and GABAgergic interneurons as well as with a reanalysis of data from published CA2 and CA1 pyramidal cells.

    I commend and congratulate the authors for this efforts here. The rodent hippocampal circuitry remains one of the most intensely investigated cortical circuit with ever increasing data on its cell types. Despite this premise, to fully leverage this immense knowledge, it requires efforts like this one presented here to catalogue and a synthetize the fragmented information across different labs and experimental conditions. In this respect the authors ongoing efforts are absolutely critical for a better understating of the relationship between cellular and circuit architecture and physiological, and perhaps ultimately behavioral, functions of the hippocampal-entorhinal circuitry.

    I think this update on the authors' continued efforts would warrant a publication. Obviously, there are many other exciting potential directions this database can be further developed by for example integrating with available information on connectivity and genetic information on hippocampal cells types, which would make the effort perhaps even more compelling to a general audience, however it is arguably beyond the scope of the current iteration of the database update.

We thank the reviewer for the positive comments and appreciation of our work. As the reviewer comments, we plan to build up from here to further integrate additional layers of information into Hippocampome.org.

Reviewer #2, Linda Katona: In this manuscript the authors present an open-science classification system they developed which enables the integration of single-cell phenotypes with the functional understanding of their complex networks. This is a very timely update to an existing online dataset (Hippocampome.org) of hippocampal and entorhinal cortical neuron types. The major breakthrough arises from data mining the current literature and mapping the in vivo oscillatory-state dependent activity patterns of single cells to Hippocampome.org cell types. Furthermore, it is very important that the authors complement gaps in our knowledge system by recording underrepresented and missing cell types under different conditions. The conclusion is that such knowledge-based explorations can promote augmented understanding of hippocampal-entorhinal function.

    This is a much awaited common framework which should permit the annotation of genetically targeted high-throughput recordings based on the much fewer but morphologically and neurochemically identified neuron types. Overall the manuscript is impressive but there are some major points that need to be addressed before it can meet publication criteria. I have also included additional minor comments to improve the presentation.

We thank the reviewer for her positive comments. See below for specific responses to your valuable comments.

    Major comments
    1.The authors demonstrate the system's successful application. Would the authors expect for it to perform just as well if the data are more similar i.e. SST+ O-LM cells vs SST+ bistratified cells vs SST+ long-range projecting GABAergic neurons? Could this be tested with some existing dataset? Have the authors considered including oscillatory state-dependent action potential autocorrelograms as categorical variables to improve on the classification?

The reviewer made an important point, which we are actually working on. We chose to present the example of PV+ interneurons because the amount of data we were able to collect so far is sufficient to demonstrate that the approach works very reliably based on the current ground truth. Classification of SST+ cells is more challenging given the poorer separation of SST+ O-LM cells versus SST+ bistratified cells for preferred theta phase. Below is an example. Note that, despite an apparent division just around unitary SWR ratio, the separation between O-LM cells (purple) and bistratified cells (red) is still unreliable given the available dataset, in the sense that k-means failed to cluster them separately. We are actively working on building a larger dataset and integrating other distinct features (including run/stop transitions, gamma locking, and possibly more). Moreover, as the reviewer herself commented, SST+ long-range projection cells should also be considered, and this requires gathering additional data. We are now working actively in this direction in combination with ultra-dense recordings using Neuropixels. Waiting for these additional results would impose an excessive delay to making the existing strong results public but, conversely, the current evidence displayed below is too preliminary and we are not sufficiently confident it will be confirmed by a more complete dataset. Based on this, we chose not to include SST+ cells at this time. The current manuscript will thus provide the proof-of-concept for PV+ cells, from where we will scale up to the next level of complexity in future papers.

Regarding autocorrelograms as categorical variables for classification, we chose not to include them because they are typically used as part of unit sorting methods, but the reviewer’s point is well taken. As commented before, we are now working in this direction by desiging multimodal classifiers operating over ultra-dense recordings. This will hopefully be part of an upcoming paper.

    2a.The authors identified gaps in the knowledge-base and have chosen appropriate recording techniques to acquire the required data to fill these gaps. However, more detail should be provided in the methods section about the analytical tools used (i.e. pairwise correlation) and please justify your choice in comparison to other multidimensional classifiers.

We appreciate the constructive criticism. We have now expanded the description of the pairwise correlation analysis in the Methods section, which also expands upon the reasoning behind our selection of analysis approach. In addition, we have performed a second form of correlation analysis, Spearman’s rank correlation coefficient, and have detailed its application, also in the Methods section.

A standard multidimensional classifier would be used to assign vectors of neuronal properties to suggested groupings or classes. However, Hippocampome.org already has clearly defined neuron types. Instead, we are interested in exploring the relationships between the neuronal properties themselves to uncover potential links between in vitro and in vivo properties. The lack of uncovered correlations suggests that in vivo properties could be useful in further identifying neuron types in extracellular in vivo recordings.

    2b.The authors should explain the categorical properties listed, especially those related to the newly introduced firing pattern phenotypes (page 32, 2nd para, Methods, Pairwise correlation analysis).

We have added more details to the Methods section about the specific categorical properties that were analyzed, taking special care to explain the categorization of the new in vivo data.

    3.The discussion is quite lengthy and speculative in places (e.g. 2nd para on page 25). Please make it more concise.

We have revised the Discussion and the particular paragraph you mention to make them more concise.

    4.I could not find this relevant publication listed in Supp. Table 2: Extended interneuronal network of the DG (Szabo et al., 2017 Cell Reports). If not there yet, it should be uploaded to the Hippocampome.org as it complements nicely the freshly acquired data.

Thank you for the suggestion. We were able to mine Szabo et al. for information about SWR ratios and DS ratios for 3 DG neuron types and have added them to the new “In vivo recordings” matrix. The other neurons that are described in the article have distinct dendritic and axonal patterns that have not been reported before, but are all single cases (N=1). As such, they do not qualify for inclusion in the “vetted” sections of Hippocampome.org yet, but we have added them to the list of On-Hold Types (<http://hippocampome.org/devur2/Help_On-hold_Types.php>).

    Minor points
    1.page 4, 1st para, Results, Mapping single-cell oscillatory dynamics to Hippocampome.org cell types    Please update your figure referencing, it should not start with a Supplementary Figure and Table in the main text.

We now call the main Fig.1 first and then refer to the Supp.Fig. and Table

    2.page 31, 1st para, Methods, Histology and immunostaining
    Multichannel fluorescence stacks were acquired... Please give details about the objectives used, the spectral channel separation (beam splitter settings), pin hole size, optical section thickness and Z stack height. Was there any post processing applied to the images (filtering, Z-projections)?

We have added the following information to the revised manuscript (see Methods).

Multichannel fluorescence stacks from recorded cells were acquired with a confocal microscope (Leica SP5) with LAS AF software v2.6.0 build 7266 (Leica) and objectives HC PL APO CS 10.0x0.40 DRY UV, HCX PL APO lambda blue 20.0x0.70 IMM UV and HCX PL APO CS 40.0x1.25 OIL UV. Pinhole was set at 1 Airy, and the following channels settings applied (fluorophore, laser and excitation wavelength, emission spectral filter): a) bisbenzimide, Diode 405 nm, 415–485 nm; b) Alexa Fluor488, Argon 488 nm, 499–535 nm; c) Rhodamine Red / Alexa Fluor594, DPSS 561nm, 571–620 nm; d) Alexa Fluor633/ Alexa Fluor647, HeNe 633 nm, 652–738 nm. Optical section intervals were of 5, 2 and 1 µm for 10x, 20x and 40x objectives, respectively. Brightness and contrast were adjusted with the ImageJ software (NIH Image). For illustration purposes, z-projections (average intensity) were made in some images. Analyses of co-location, cell position and/or quantifications were always made at one confocal plane.

    3.Missed citations/referencing:
    -page 3: Single-cell phenotyping based on firing behavior in vivo has provided a large body of evidence to characterize microcircuit operation [7-10, 75, 21, 25].

Thanks, we added these.

    -page 4: By integrating in vivo functional PoKs (e.g. preferred theta phases and SWR-associated firing dynamics) into Hippocampome.org, we extended the previous classification of certain neuron types using novel information on oscillatory dynamics [19].

Thanks, we added this.

    -page 5: to the Collateral-related cell superfamily (Supp. Table 1)

Because we now call the table just before this sentence, we felt that now this is no longer required.

    -page 5: Neurons morphologically identified and/or tested against a battery of neurochemical markers enabled their assignment to specific Hippocampome.org types (Fig. 1)

Thanks, we added this.

    -page 7: For instance, GABAergic CA1 bistratified cells preferentially fire during the ascending ripple phase (Fig.1A, [21]), while O-LM cells tend to decrease their firing rate during SWR as compared with baseline (Fig.1B, [7]); but see also [25, 7] for heterogeneous participation of these cells during a variety of SWR.

Thanks, we added these.

    -page 8: most CA1 pyramidal cells fire near the theta trough (Fig.1D, [ref?]). Finally, single-cell firing dynamics during gamma (25-90 Hz) and/or so-called epsilon (90-130 Hz) oscillations were reported in a minority of cases (Fig.1E) [ref?]

Thanks. We added refs 6 & 28 for F1D and 27 & 29 for F1E.

Reviewer #3, Szabolcs Káli: The manuscript describes a rather complex body of work whose primary purpose is to provide curated and consolidated information about the firing properties of hippocampal and entorhinal cortical neurons in relation to the main hippocampal population activity patterns. This information is important from several different aspects: it allows the identification of cell types from extracellular recordings in vivo (as demonstrated by the authors), defines fundamental constraints for the validation of realistic network models, and provides a conceptual framework for theories and further experimental studies of hippocampal network function. Results presented in this paper include the curation of data from the experimental literature, which was a highly nontrivial task in itself despite the relatively limited number of relevant articles, due to the large heterogeneity of experimental preparations and recording methods; new as well as previously published but reanalyzed experimental data from several different areas, cell types, and conditions; and, importantly, the integration of this new knowledge into the online database Hippocampome.org.

We thank the reviewer for his positive comments and the many suggestions, which we address one by one below.

    While the overall aim of the study is clearly of high importance, the execution of the work (especially the experimental part) is of high quality, and most details are presented clearly, the coherence of the manuscript could be improved substantially. I sometimes had the impression of reading two separate, but interleaved papers - one about how information about the firing of hippocampal neurons relative to network events was collected and used, and another one about the various fascinating but sometimes loosely related findings from a variety of new experiments in vivo. In my view, this issue stems from the dilemma that, on the one hand, many of the new experimental findings would deserve a more thorough description and discussion but, on the other hand, some of the existing discussion already distracts the reader from what is declared to be the main topic of this paper.

We have polished the writing to improve the coherence of the manuscript. Specifically: (i) we moved the most technical description of how we did the curation from the Results to the Methods; (ii) we reduced the overall length of narrative providing series of examples to sharpen, rather than drag, the most novel discoveries; (iii) we added a new Table 3 and accompanying paragraph at the beginning of Discussion to highlight the clear insights into what we found from the synergy of data curation and additional experiments. We hope the new version reads better and more successfully integrates the synergistic contributions of lit mining and new experiments. Indeed the main value of our work is at the interface between filling experimental gaps and their integration in knowledge-based databases to accelerate understanding of hippocampal-entorhinal function. As we summarize in the abstract, our approach was to identify missing and unresolved pieces of knowledge and to complement the dataset with our own new data. To help readers with an appropriate context, we have also added a sentence in the last paragraph of the Introduction to explain the synergistic integration of literature mining and new experimental evidence.

    I commend the authors for making available for review a new version of Hippocampome.org that includes the new data and other improvements associated with this paper. I spent some time exploring this new version of the website, and I would like to mention some issues that the authors may want to address before the paper is published and the new version becomes public.
    First, I was unable to locate some of the new data from the current manuscript on Hippocampome.org. In particular, it seems to me that the data on in vivo membrane properties (presented in Table 1 of the paper) has not been added to the appropriate part of Hippocampome.org.

We thank the reviewer for pointing us to this detail. The new in vivo membrane properties are available when the user opts to expand the new In vivo recordings matrix, such that the “Other” column becomes 11 newly revealed columns. The check box used to expand the matrix has been relocated on the screen, made larger, and encircled to help draw attention to it.

    Second, while the creation of new subcategories for certain cell types (e.g., deep and superficial CA1 pyramidal neurons) is certainly justified by the available data (both in the current manuscript and other recent papers), this creates various challenges for the database and the user interface, some of which appear to be currently unsolved. For instance, the various tables of cellular properties (e.g., the "electrophysiology matrix") now list CA1 Superficial PCs and CA1 Deep PCs instead of CA1 PCs, but the table entries (and associated references) for both new cell types were apparently copied over from the earlier CA1 PC entry, and therefore give identical values for the two new subtypes. This gives the false impression that (at least according to our current knowledge) these two cell types have identical intrinsic physiological properties, even though the current manuscript (as well as other papers) now provide ample evidence to the contrary. In my opinion, the creation of new subtypes should be treated consistently in every part of the database - although I assume that this is a difficult task which may require manual re-evaluation of relevant parts of the literature. In addition, a way should be found to include and clearly display data that are specifically about the newly created subtypes, but also data which are associated with the more general cell type (i.e., where the subtype is unknown).

We acknowledge this useful suggestion. We have modified several of the matrices to now include a generic CA1 Pyramidal cell type, along with the Superficial and Deep subtypes. The latter two are indented and colored to clearly separate them and their properties from those of the generic type. The matrices that have been modified are the morphology, markers, membrane biophysics, connectivity, and in vivo recordings matrices.

    Third, the ability to filter data points according to a variety of factors is a great new feature which considerably enhances the usability of the database, especially given the substantial differences that are sometimes observed between experimental conditions. However, this feature does not currently seem to work as expected. For example, if I select a combination of factors (conditions) for which there are currently no data available (or if I simply deselect all factors) and press the "update" button, the table gets updated, but there are still values shown in every cell of the table (except in a few that are always empty) - although when I hover the cursor over these entries, the system (correctly) shows the number of relevant sources and measurements as 0. I also suggest that, when this feature works correctly, the functionality should eventually be extended to all parts of the database (although this work is clearly beyond the scope of the current manuscript, and  should not affect its publication).

We have fixed the functionality of the metadata selectors and display, so that no data appears in the new “In vivo recordings” matrix when the user deselects all of the metadata.

Similarly, I found the graphical representation of the data in Figure 5A to be very informative and easier to digest than the table format in Figure 5B that is currently included in Hippocampome.org, so it may be worth considering the addition of such as graphical representation (possibly in an interactive format) to the website.

We have added a downloadable JPG version of Figure 5A that is linked to the new “In vivo recordings” matrix page. We have determined that creating an interactive version of the figure is not practical, as it will likely include installing additional plotting libraries on our server, working with a new plotting language to create a specialized interactive type of plot, and programming database access methods to dynamically retrieve relevant data for the plot. We will, however, consider it as a project moving forward for the next version of Hippocampome.org

    Although the reasoning in the paper is generally quite sound, I cannot agree with the conclusions of the paragraph in lines 410-416. Here, after observing that the newly added in vivo firing properties of neurons do not show significant pairwise correlations with any of the previously described (in vitro) characteristics, the authors conclude that "these in vivo properties are largely independent and could thus add classificatory power when combined with traditional in vitro characterization." However, the fact that individual (linear) correlations with individual variables are not statistically significant does not imply that the new features could not be predicted (possibly with high confidence) by some (potentially nonlinear) combination of all the in vitro features. In fact, it would be surprising if the new features and the older ones were truly independent as both of these are shown to be strongly dependent on the cell type. Combinations of morphological, electrophysiological, and molecular features have proved to be good predictors of cell type (as demonstrated beautifully by Hippocampome.org), and the cell type is a good predictor of in vivo firing behavior (as shown by the data summarized in the current manuscript). On the other hand, I agree with the claim that the newly added features may be uniquely useful for the categorization of neurons in the context of in vivo ensemble recordings where the majority of the other features that are traditionally used for classification are typically not available.

We appreciate this perspective and have now integrated it into the revised manuscript. Specifically, we have expanded upon the Results, making the point that the in vivo data are still too sparse to reach a definitive conclusion from such an analysis, although they suggest that the in vivo data can enhance the utility of in vitro classification. We also added a section to the Discussion concerning the conclusions of our correlation analysis. We point out, as the Reviewer suggests, that the results are applicable only to a linear or monotonic relationship between the in vivo and in vitro data and that a future analysis (when sufficient data are available) likely would prove beneficial in uncovering a non-monotonic link between the two forms of data.

    Finally, I suggest that the manuscript may benefit from some thorough proofreading as I found several typos as well as minor errors in grammar and usage throughout the text.

Thanks. We carefully proofread the manuscript for optimal readability.

    Minor issues:
    -        I think that intracellular recordings using sharp micropipettes are more commonly referred to as "sharp electrode recordings" rather than "sharp recordings"

Thanks. We corrected this.

    -        the reference "Rees et al., 2017" on line 467 appears to be incorrectly formatted, and is not included in this form in the list of references (although there is an entry for Rees et al., 2016)

Thanks. We corrected the inconsistency.
